# Tetrahydrobiopterin: Beyond Its Traditional Role as a Cofactor

**DOI:** 10.3390/antiox12051037

**Published:** 2023-05-03

**Authors:** Tuany Eichwald, Lucila de Bortoli da Silva, Ananda Christina Staats Pires, Laís Niero, Erick Schnorrenberger, Clovis Colpani Filho, Gisele Espíndola, Wei-Lin Huang, Gilles J. Guillemin, José E. Abdenur, Alexandra Latini

**Affiliations:** 1 Laboratório de Bioenergética e Estresse Oxidativo—LABOX, Departamento de Bioquímica, Centro de Ciências Biológicas, Universidade Federal de Santa Catarina, Florianópolis 88037-100, SC, Brazil; tuany.eichwald@posgrad.ufsc.br (T.E.); lais.niero@grad.ufsc.br (L.N.); clovis.l.c.filho@grad.ufsc.br (C.C.F.); gisele.espindola@ebserh.gov.br (G.E.); 2 Laboratory for Energy Metabolism, Division of Metabolic Disorders, CHOC Children’s Hospital, Orange, CA 92868, USA; wei.lin.huang@choc.org (W.-L.H.); jabdenur@choc.org (J.E.A.); 3 Neuroinflammation Group, Department of Biomedical Sciences, Faculty of Medicine and Health Sciences, Macquarie University, Sydney, NSW 2109, Australia

**Keywords:** antioxidant, neopterin, sepiapterin, mitochondrial enhancer, memory, inflammation, oxidative stress

## Abstract

Tetrahydrobiopterin (BH4) is an endogenous cofactor for some enzymatic conversions of essential biomolecules, including nitric oxide, and monoamine neurotransmitters, and for the metabolism of phenylalanine and lipid esters. Over the last decade, BH4 metabolism has emerged as a promising metabolic target for negatively modulating toxic pathways that may result in cell death. Strong preclinical evidence has shown that BH4 metabolism has multiple biological roles beyond its traditional cofactor activity. We have shown that BH4 supports essential pathways, e.g., to generate energy, to enhance the antioxidant resistance of cells against stressful conditions, and to protect from sustained inflammation, among others. Therefore, BH4 should not be understood solely as an enzyme cofactor, but should instead be depicted as a cytoprotective pathway that is finely regulated by the interaction of three different metabolic pathways, thus assuring specific intracellular concentrations. Here, we bring state-of-the-art information about the dependency of mitochondrial activity upon the availability of BH4, as well as the cytoprotective pathways that are enhanced after BH4 exposure. We also bring evidence about the potential use of BH4 as a new pharmacological option for diseases in which mitochondrial disfunction has been implicated, including chronic metabolic disorders, neurodegenerative diseases, and primary mitochondriopathies.

## 1. Tetrahydrobiopterin Biosynthesis

**Figure 1:**
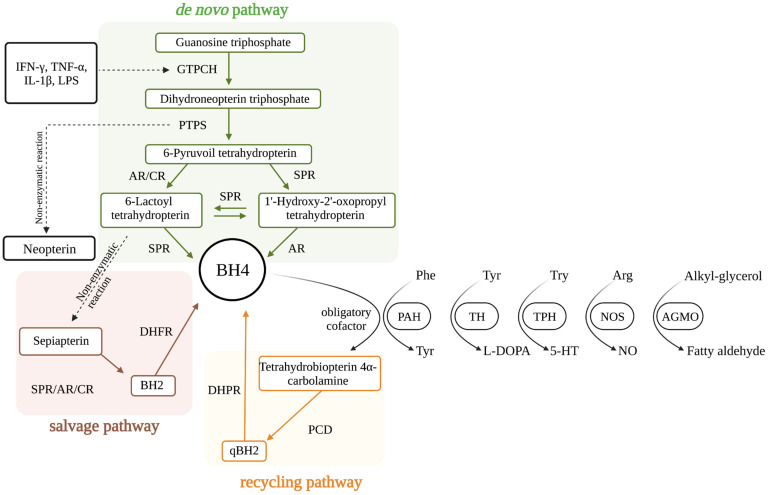
Metabolic pathways involved in the biosynthesis of tetrahydrobiopterin (BH4). ***De novo* pathway:** guanosine triphosphate cyclohydrolase (GTPCH), 6-pyruvolyl tetrahydropterin synthase (PTPS), and sepiapterin reductase (SPR) transform guanosine triphosphate into BH4. The last enzymatic step catalyzed by SPR can be overcome by the unspecific reductases aldose reductase (AR) and carbonyl reductase (CR). This is possible due to an active interaction between the de novo and the salvage pathways, where AR, CR, and/or SPR utilize intermediates of the de novo pathway to generate the key intermediate of the salvage pathway, sepiapterin. **Salvage pathway:** sepiapterin is transformed into dihydrobiopterin (BH2), then reduced to BH4 by dihydrofolate reductase (DHFR). **Recycling pathway:** Pterin-4-alpha-carbinolamine dehydratase (PCD) transforms BH2 in dihydrobiopterin quinoid (qBH2), which is reduced back to BH4 by dihydropteridine reductase (DHPR). BH4 is an obligatory cofactor for the activity of the aromatic amino acid hydroxylases, phenylalanine hydroxylase (PAH), tyrosine hydroxylase (TH), and tryptophan hydroxylase (TPH), for all isoforms of nitric oxide synthase (NOS), and for alkylglycerol monooxygenase (AGMO). Abbreviations: IL-1β: interleukin-1 beta; TNF-α: tumor necrosis factor-alpha; INF-γ: interferon-gamma; IL-6: interleukin-6; LPS: lipopolysaccharide.

6R-L-erythro-5,6,7,8-tetrahydrobiopterin (BH4) is an enzyme cofactor that is essential for the synthesis of monoamine neurotransmitters, for the metabolism of phenylalanine (Phe) and lipid esters, and for the production of nitric oxide (NO) [1]. The intracellular concentration of BH4 is strictly maintained at low levels by three finely tuned biosynthetic pathways: the de novo, recycling, and salvage pathways (Figure 1).

The synthesis of BH4 is a complex metabolic pathway and involves several metabolites, including 7,8-dihydrobiopterin (BH2), which is the oxidized form of BH4, neopterin that has been associated with inflammatory conditions for decades, sepiapterin that has recently been identified as another precursor of BH4, and BH4 itself. The de novo BH4 pathway reduces guanosine triphosphate (GTP) into BH4 by the successive action of three enzymes: GTP cyclohydrolase I (GTPCH), 6-pyruvoyl-tetrahydrobiopterin (PTPS), and sepiapterin reductase (SPR). In the absence of SPR, non-specific reductases (aldose reductase (AR) and carbonyl reductase (CR)) can also generate BH4. SPR, AR, and CR also participate in the salvage pathway, which utilizes a metabolic intermediate previously formed in the de novo pathway, 6-pyruvoyl-tetrahydrobiopterin, to generate a non-stable intermediate that non-enzymatically forms sepiapterin. Sepiapterin is then metabolized by SPR or CR into BH2, which is further transformed into BH4 by the action of dihydrofolate reductase (DHFR). Finally, in the recycling pathway, after BH4 is used as a mandatory enzyme cofactor and transformed into quinonoid dihydrobiopterin (qBH2) by pterin 4α-carbinolamine dehydratase (PCD), dihydropteridine reductase (DHPR) acts on the qBH2 intermediate to regenerate BH4 [1,2] (Figure 1).

Under physiological conditions, GTPCH is the rate-controlling enzyme in the BH4 pathway [1]. GTPCH is encoded by *GCH1*, which is positively regulated by a variety of inflammatory and oxidant mediators, including lipopolysaccharide (LPS), interferon gamma (IFN-γ), interleukin 1 beta (IL-1β) [3], tumor necrosis factor alpha (TNF-α) [4], hydrogen peroxide [5], and others (Figure 1). During inflammation *GCH1* is markedly upregulated, however, levels of other constitutive downstream enzymes that mediate the de novo pathway (PTPS and SPR) are only slightly increased, leading to a *pseudo* metabolic blockage and a consequent accumulation of neopterin [6]. Indeed, neopterin has long been used as a sensitive biomarker for innate immune system activation for multiple acute and chronic conditions [7,8].

GTPCH is also regulated by allosteric feedback via BH4 and Phe, which interact with the GTPCH feedback regulatory protein (GFRP) [9]. GFRP acts as both a positive and negative regulator of GTPCH [10]. High Phe levels are known to stimulate GTPCH activity, resulting in activation of the de novo pathway and BH4 production. When intracellular levels of BH4 are sufficient for proper metabolism, GTPCH activity is negatively modulated [11].

## 2. Levels of BH4 and Related Metabolites in Biological Fluids

Pediatric and adolescent reference values for the biological fluid levels of neopterin, biopterin (fully oxidized BH4), and BH4 are shown in Table 1 [12]. The table shows that the levels of pterins in cerebrospinal fluid (CSF) and urine decrease with age (Table 1), but no correlation with age is seen for neopterin and biopterin levels in serum and dried blood spot samples. These trends contrast with those observed in adults, where serum/plasma and urine neopterin levels correlate with age, but not sex [13] (Table 2). Neopterin levels are highest in the elderly, probably due to chronic inflammation [14]. The renal clearance of neopterin is similar to that of creatinine, therefore levels cannot be measured in patients with impaired renal function [15,16].

Neopterin concentrations in CSF are somewhat lower than those in serum or plasma in adults [15] (Table 2). Similarly, neopterin concentrations are elevated in the blood, but not the CSF of individuals with neurological neuroinflammatory chronic conditions who also have normal blood brain barrier (BBB) function [16]. Neopterin present in the CSF is likely to have a central origin, since it can cross the BBB only at a very low quotient (1/40) [7,15,16]. Additionally, our group recently demonstrated that neopterin is secreted by human brain cells, neurons, astrocytes, and microglia after LPS or IFN-g challenge [17].

Unfortunately, there are no defined reference levels for BH4 in the adult population, probably due to the methodological difficulties in measuring the metabolites in the pathway. Moreover, sample processing for BH4 quantification requires acidic treatment and/or the addition of antioxidants, since the pterin is extremely sensitive to pH, and it oxidizes into BH2 at a pH higher than 4. In addition, processed samples need to be frozen immediately and must always be protected from direct light [18,19]. In contrast, neopterin is a stable molecule that does not require excessive processing and it is stable in urine samples left at room temperature or 4 °C for days, or frozen for months. Because of the lack of defined reference levels for BH4, it is appropriate to have the controls measured in parallel when measuring BH4 in biological samples, like serum/plasma and urine from different cohorts. 

## 3. BH4 as an Enzyme Cofactor

**Figure 2:**
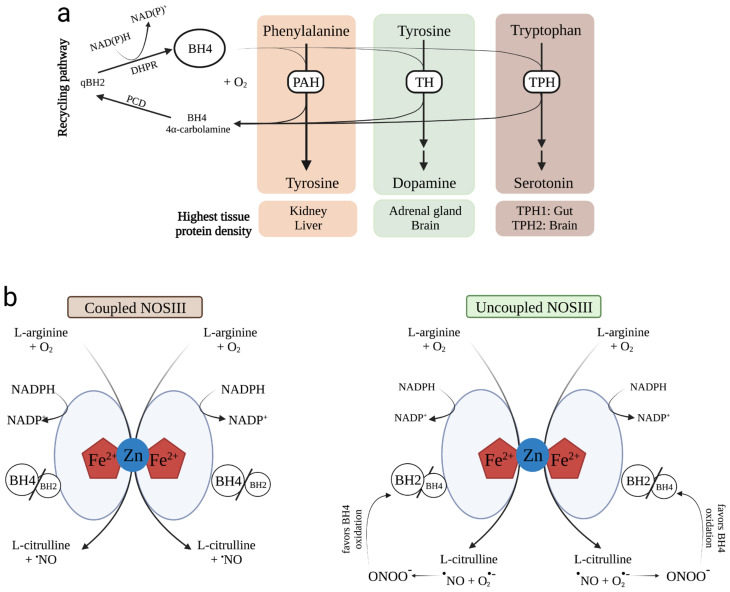
(**a**) Phenylalanine (PAH), tyrosine (TH), and tryptophan (TPH) hydroxylases are dependent on tetrahydrobiopterin (BH4) for their biological activities. Due to its cofactor function, BH4 is required to synthesize tyrosine from phenylalanine, dopamine from tyrosine, and serotonin from tryptophan. After functioning as a cofactor, BH4 is regenerated via the BH4 recycling pathway. Dihydrobiopterin quinoid (qBH2) is then formed by pterin 4α-carbinolamine dehydratase (PCD) and dihydropteridine reductase (DHPR). Protein levels are highest in kidney and liver for PAH; in the adrenal gland and brain for TH; and in the gut and brain for TPH1 and TPH2, respectively. (**b**) Tetrahydrobiopterin (BH4) availability is compromised by the uncoupling of nitric oxide synthase III (NOSIII). *Coupled NOSIII*: L-arginine and molecular oxygen (O_2_) are the substrates for NOS III to produce _L_-citrulline and nitric oxide (NO). The reaction requires as cofactors, the mandatory presence of O_2_ and appropriate levels of BH4, as well as reduced nicotinamide adenine dinucleotide phosphate (NADPH). Coupled NOSIII is presented as a heme-containing dimer stabilized by zinc (Zn). Zn is responsible for connecting two NOSIII monomers at the heme group site. BH4 exerts structural and biochemical functions, helping to stabilize the dimer and controlling the coupling of O_2_ to _L_-arginine oxidation. When BH4 levels become deficient and the levels of its oxidized product dihydrobiopterin (BH2) increase (reduced BH4/BH2 ratio), the NOSIII-catalyzed reaction results in the formation of _L_-citrulline, NO, and superoxide anion (O_2_^−^). O_2_^−^ is a very reactive radical species that produces peroxynitrite (ONOO^−^), a highly reactive oxidant that favors BH4 oxidation. Abbreviations: Fe^2+^: iron; NADP^+^: nicotinamide adenine dinucleotide phosphate. Adapted from Kim and Han, 2020 [53]. The symbol “^•^” is used to indicate the presence of an unpaired electron in ^•^NO (nitric oxide), O_2_^•^ (superoxide anion), and ONOO^−^ (peroxynitrite).

BH4 is known widely to act as an enzyme cofactor for a select few enzymatic reactions, which involve Phe hydroxylase (PAH), Tyr 3-hydroxylase (TH), and tryptophan-5 hydroxylase (TPH) (Figure 2a), all isoforms of NO synthases (NOS I, II and III) (Figure 2b), and alkylglycerol monooxygenase (AGMO).

### 3.1. PAH

The transformation of Phe into the semi-essential amino acid L-tyrosine (Tyr) is catalyzed by PAH (EC: 1.14.16.1) [20]. PAH was the first enzyme to be discovered to have BH4 as a mandatory cofactor for the hydroxylation of Phe. PAH contains a non-heme non-iron–sulfur iron (Fe^2+^) in the active site, where BH4 and molecular oxygen bind to promote hydroxylation of the amino acid. As a result, Tyr is formed, and BH4 is transformed into qBH2, which will be used by PCD and DHPR to regenerate BH4 in the recycling pathway [1]. According to the human protein atlas (humanproteinatlas.org, accessed on 15~March 2023), PAH distribution is tissue specific, with highest levels in liver, in agreement with its participation in the hepatic synthesis of Tyr [21].

PAH genetic deficiency leads to hyperphenylalaninemia (HPA) and to the most common inborn error of amino acid metabolism, phenylketonuria (PKU) [22]. Both conditions, HPA and PKA, can be identified by measuring the Phe and Phe/Tyr ratio during routine neonatal screening [23]. Mild PAH deficiencies result in modest elevations of Phe (below 360 umol/L) and do not require any treatment. However, severe PAH deficiency leads to PKU, characterized by the persistent accumulation of Phe greater than 360 mmol/L, which becomes toxic to the brain and impairs cognitive development [21]. The clinical symptoms of untreated PKU include intellectual disabilities, microcephaly, seizures, tremors, ataxia behavioral abnormalities, psychiatric symptoms, and decreased skin and hair pigmentation. Affected individuals also show decreased production of myelin, as well as dopamine, norepinephrine, and serotonin [21]. Treatment consists of a Phe-restricted diet and should be initiated as soon as possible after birth in order to normalize blood concentrations of Phe and Tyr, and to prevent cognitive deficits [21]. Some patients respond to additional treatment with the PAH cofactor BH4, which allows for an increase in protein intake or even liberalization of the diet [24]. More recently, treatment with an alternative enzyme, phenylalanine ammonia lyase (PAL), that deaminates Phe to cinnamic acid and ammonia has shown very promising results [25]. 

Another form of HPA was identified in a group of patients with increased Phe levels, who showed developmental regression, abnormal movements, and seizures after a few months of normal early development. This unusual presentation was called “atypical or malignant HPA” [26]. It was later established that the elevation in Phe in these patients was caused by deficient BH4 concentrations, establishing the fact that pterin can act as an enzyme cofactor donating electrons (Figure 2a), and as a pharmacological chaperone. In fact, it was shown that BH4-mediated structural stabilization restored the dysfunctional PAH monomer binding to the PAH active site, leading to enhanced PAH activity [27]. Currently, children identified as having increased Phe levels in their newborn screening sample undergo additional testing, including the measurement of BH4 and BH4-related metabolites concentrations, enzymatic activity for enzymes involved in BH4 synthesis, and/or molecular testing [28]. In some centers, a BH4-loading test is also performed measuring Phe levels at different times after a single dose of sapropterin dihydrochloride (commercial BH4 analogue) (see Section 4 below) [28]. 

### 3.2. TH

The first enzymatic step for dopamine synthesis is catalyzed by TH (EC: 1.14.16.2), which transforms Tyr into L-3,4-dihydroxyphenylalanine (L-DOPA) [29] (Figure 2a). Similar to PAH, TH uses molecular oxygen to hydroxylate its substrate in a reaction that requires finely tuned intracellular BH4 levels [30]. Electrons transferred from BH4 interact with a non-heme, non-iron–sulfur iron (Fe^2+^) in the active site during hydroxylation. TH can be regulated by phosphorylation at a serine residue (e.g., Ser^8^, Ser^19^, Ser^31^, and Ser^40^) present in the active site, which rapidly increases activity levels (for a review see [31]). TH is mostly expressed in brain tissue, primarily in the cytosol, but is also associated with neurotransmitter-containing vesicles in the plasma membrane (humanproteinatlas.org, accessed on 15 March 2023). 

Deficient TH activity results in impaired dopamine synthesis and lower concentrations of its catabolite homovanillic acid (HVA) [32]. Any change in TH expression or activity directly impacts dopamine neurotransmission, and a TH deficiency may lead to the development of idiopathic Parkinson’s disease (PD). Indeed, postmortem human striatum from patients with PD shows reduced TH levels [33]. 

Hereditary TH deficiency causes L-DOPA–responsive dystonia (DRD), an autosomal recessive movement disorder that can present in childhood or adolescence, with different degrees of severity, and is characterized by progressive dystonia with diurnal variation, and response to treatment with L-DOPA [34,35]. Pharmacological treatment with L-DOPA and/or dopamine agonists is effective, but prognosis depends on symptom severity and how early treatment is initiated, ranging from completely resolved symptoms to persistent dystonia and rigidity [36]. Supplementation with BH4 has recently been shown to restore dopamine content and TH activity in a rodent model of PD [37].

It is important to note, that there are other genetic conditions that can mimic DRD but are not caused by defects in TH, therefore molecular confirmatory testing is important to confirm the diagnosis for TH deficiency [38]. 

### 3.3. TPH

Tryptophan (Try) is transformed into 5-hydroxytryptophan by the biological activity of TPH (EC: 1.14.16.4) in the presence of molecular oxygen and BH4. Similar to the other two BH4-dependent hydroxylases, TPH hydroxylates its substrate through the interaction of molecular oxygen with a non-heme, non-iron–sulfur iron (Fe^2+^) in the active site. BH4 facilitates the activation of molecular oxygen and leaves the reaction as the intermediate pterin 4a-carbinolamine, which is reduced back to BH4 by PCD and DHPR activities in the BH4 recycling pathway [39] (Figure 2a). The activity of THP can be enhanced by the presence of Try and BH4, or by phosphorylation. For example, THP1 is a target of protein kinase A, while THP2 can be phosphorylated by calmodulin-dependent protein kinase II [40]. Increased TH activity may increase the availability of serotonin, which has antidepressant effects [41]. On the contrary, impaired TH activity, e.g., following exposure to a selective and irreversible inhibitor of THP, such as *p*-chlorophenylalanine, may precipitate depression in animals.

TPH has two isoforms. The TPH1 isoform is mainly expressed in peripheral tissues, where it regulates vasoconstriction and the control of immune responses. Previous research has shown that 90% of circulating serotonin is produced by the gut [42]; however, THP1 is also expressed in the skin, pineal gland, and the central nervous system. TPH2 is present exclusively in the brain and is involved in the central control of food intake, sleep, and mood [43]. 

No clear association between genetic defects in TPH and human disease has been found to date. 

### 3.4. NOS

All NOS isoforms (NOSI, II, and III, formerly called neuronal, inducible, and endothelial NOS) (EC: 1.14.13.39) catalyze the conversion of _L_-arginine (Arg) into _L_-citrulline plus nitric oxide [44]. All NOS isoforms have similar homodimeric structures, where each monomer has an N-terminal oxygenase domain with binding sites for Arg, heme, and BH4. Additional cofactors flavin adenine dinucleotide (FAD) and flavin mononucleotide (FMN) bind to the reductase domain, which donates electrons from reduced nicotinamide adenine dinucleotide phosphate (NADPH) to the oxygenase domain [45]. BH4 is not involved in activation of the oxygen as it is in the BH4-dependent hydroxylases, but supplies one electron to the reaction, yielding a protonated trihydropterin radical [46]. This radical is then regenerated to BH4, while bound to the enzyme by a process that involves the transferring of an electron from NADPH via the FAD and FMN sites of the reductase domain (Figure 2b). BH4 presumably also supplies a proton to the NOS active site via a proton bridge [1]. 

BH4 also enhances the structural functions of NOS during the synthesis of NO. BH4 allows dimer stabilization and dimer formation, protects against proteolysis, and increases Arg binding [47]. Under these physiological conditions, NOS is coupled, and NO is formed. However, when BH4 is consumed, e.g., during oxidative stress, and the BH4/BH2 ratio is compromised, NOS is uncoupled, and in addition to NO production, the anion superoxide radical is formed. As a result, a short-lived and reactive biological oxidant called peroxynitrite (ONOO^−^) is generated from the diffusion-controlled reaction of the free radicals, superoxide and NO [48].

NOSI and NOSIII are constitutively expressed, and their activity is regulated by calcium and calmodulin. NOSI is present in brain tissue; however, the protein has also been identified by immunohistochemistry in the spinal cord, the sympathetic ganglia and adrenal glands, the peripheral nitrergic nerves, the epithelial cells of various organs, kidney macula densa cells, pancreatic islet cells, and the vascular smooth muscle [49]. The largest source of NOSI (in terms of tissue mass) in mammals is skeletal muscle [49]. NOSI has been implicated in modulating learning, memory, and neurogenesis due to the ability to generate the gas retrograde neurotransmitter NO [50]. In this scenario, BH4 has been shown to facilitate learning and memory by supporting NO production in rodents, through the activity of NOSI [18].

NOSIII is mostly expressed in endothelial cells, but has also been detected in the heart, platelets, brain, placenta, and kidney [49]. NOSIII dilates all types of blood vessels by stimulating soluble guanylyl cyclase and increasing the levels of cyclic GMP in smooth muscle cells [51].

NOSII is the only inducible NOS whose expression can be induced by bacterial LPS, cytokines, and other agents. Although identified primarily in macrophages, the expression of the enzyme can be stimulated in virtually any cell or tissue. Once expressed, NOSII is constantly active and is not regulated by calcium, as are NOSI and NOSIII [49]. When present in immune cells, NOSII produces large quantities of NO, with de novo synthesis of BH4 (mandatory NOS cofactor) required for sustained enzyme activity (Figure 1). High levels of NO produced by activated macrophages may not only be toxic to undesired microbes, parasites, or tumor cells, but also compromise cell viability and homeostasis, due to the high reactivity of NO towards protein-bound iron. NO can inhibit key enzymes that contain iron in their catalytic centers, including complexes of the respiratory chain, as well as enzymes involved in the Krebs cycle and in DNA, leading to DNA oxidation and fragmentation [52].

### 3.5. AGMO

The cleavage of an ether bond of free alkylglycerols and lyso-alkylglycerophosphocholines or lyso-alkylglycerophosphoethanolamines is catalyzed by AGMO (EC: 1.14.16.5) in the presence of molecular oxygen and BH4. AGMO is localized in the membrane of the endoplasmic reticulum (www.proteinatlas.org, accessed on 15 March 2023). Although the highest expression occurs in the liver and white adipose tissue, there is some brain regional specificity of the enzyme, which seems to be essential for proper brain function. 

AGMO was first described in 1964 [54] and it remained for several decades as an orphan enzyme, until the encoding gene, *TMEM195*, was discovered [55]. *TMEM195* is thought to play a role in neurodevelopmental disorders, such as autism spectrum disorders (ASD) [56,57,58]. The relationship of *TMEM195* with the BH4 metabolic pathway is unknown; however, reduced levels of BH4 have been found in the urine and CSF of children with ASD [59]. Moreover, a clinical trial demonstrated that treatment with 20 mg/kg/day sapropterin dihydrochloride significantly improved social awareness, mannerisms, hyperactivity, and inappropriate speech, compared to a placebo in ASD affected individuals [60].

## 4. Hereditary Deficiencies of BH4 

BH4 deficiencies are a group of rare inherited neurological disorders caused by mutations in genes encoding enzymes involved in BH4 synthesis (Figure 1). The deficiencies are mainly characterized by neurotransmitter dysfunction, with or without HPA, which nowadays is typically identified at the newborn screening [61]. Neurological alterations become clear and evident at the median age of four months, and symptoms can include poor suction, impaired tone, brain atrophy, and microcephaly [62]. Early diagnosis is essential for the initiation of therapies aimed at preventing physical and mental damage.

The overall frequency of BH4 deficiencies remains to be established. The average incidence of all types of HPA in Europe has been estimated at 1:10,000 [63], with BH4 deficiencies responsible for 1 to 2% of these cases. PTPS deficiency accounts for 54% of these cases, while defects in DHPR, GTPCH, and PCD account for 33%, 4%, and 5%, respectively [64].

### 4.1. GTPCH Deficiency

GTPCH deficiency is a rare form of HPA, and 37 cases have been described in the International Database of Tetrahydrobiopterin Deficiencies (BIODEF) (biopku.org, accessed on 15 February 2023). Mutations in *GCH1* can occur in recessive (AR-GTPCH; OMIM #233910) or dominant forms (AD-GTPCH; OMIM #600225). Both, the AR-GTPCH and AD-GTPCH forms show monoamine neurotransmitter abnormalities, but only the former is accompanied by HPA [65]. In AR-GTPCH, neopterin values are usually very low, ranging from 0.09 to 0.20 mmol/mol creatinine in the urine and from 0.05 to 3.0 nmol/L in the CSF. The reduced levels of neopterin are proportional to the compromise of GTPCH activity and BH4 synthesis. Indeed, HPA is markedly reduced after a BH4 loading test (BH4 20 mg/kg) [28]. Clinical presentation of GTPCH deficiency (AD or AR forms) is characterized by diurnal fluctuation of the symptoms, with aggravation in the evening. The most frequent symptoms include hypertonia, dystonia, gait difficulties, and hyperreflexia [66]. AR-GTPCH-affected individuals present more prominent developmental delay, and global dystonia. Treatment involves a Phe-restricted diet accompanied by oral administration of sapropterin dihydrochloride (BH4 supplementation), L-DOPA/decarboxylase inhibitor, 5-hydroxytryptophan, and folinic acid (Table 3) [67]. Treatment for the AD form of GTPCH deficiency includes the restitution of BH4, via oral administration of sapropterin dihydrochloride, and a L-DOPA/decarboxylase inhibitor (Table 4) [67]. 

### 4.2. PTPS Deficiency

The deficiency of PTPS (OMIM #261640) is the most frequent and heterogeneous of all BH4 hereditary defects. At the time of submission of this manuscript, 735 cases had been reported in the BIODEF: 168 with typical severe form, 35 with atypical mild peripheral form, and only 2 with transient presentation (biopku.org, accessed on 15 March 2023). Almost two hundred genotypic variants have been described for the *PTPS* gene. Neopterin values in PTPS deficiency are very high when compared to controls (Table 1), ranging from 4.95 to 51.16 mmol/mol creatinine in the urine and 11 to 449 nmol/L in the CSF. The BH4 loading test shows a reduction in plasma Phe concentrations after the administration of 20 mg/kg BH4 [28].

Individuals affected by the typical form of the disease represent 80% of all cases and have more pronounced symptoms, with marked impairment in neurophysiological development, truncal hypotonia, increased appendicular tone, global dystonia, loss of head control, bradykinesia, swallowing difficulties, somnolence and hyperthermia, among others [62]. The atypical form is also known as the peripheral form, due to the presence of normal levels of neopterin and neopterin/BH4 ratio in the CSF, less significant HPA, and minor changes in the neurotransmitter levels, which predict excellent prognosis with normal neurological development [68]. 

The treatment of PTPS deficiency can include a Phe-restricted diet, oral sapropterin dihydrochloride (BH4 supplementation) combined with neurotransmitter precursors (L-DOPA/decarboxylase inhibitor), 5-hydroxytryptophan, and folinic acid (Table 3). 

### 4.3. DHPR Deficiency

The deficiency of DHPR (OMIM #261630) is an autosomal recessive genetic defect in the *QDPR* gene that affects the BH4 recycling pathway. Out of the 303 cases listed in the BIODEF so far, 267 presented the severe form, 12 the atypical mild form, and 21 with an unknown subtype (biopku.org, accessed on 15 March 2023). 

DHPR deficient individuals present normal or slightly increased neopterin values, ranging from 0.48 to 23.23 mmol/mol creatinine in the urine and from 11 to 70 nmol/L in the CSF (See Table 1 and Table 2 for comparison with normal levels). The BH4 loading test shows a reduction in plasma Phe concentrations after the administration of 20 mg/kg BH4 [28].

Clinical presentation of DHPR deficiency is variable, but the severe forms are among the most devastating of all the BH4 deficiencies. Symptoms include progressive mental retardation secondary to extensive neuronal loss, abnormal vascular proliferation in the brain and basal ganglia calcification, and sudden death [69,70,71]. Treatment includes the administration of neurotransmitter precursors L-DOPA/decarboxylase inhibitor, 5-hydroxytryptophan, and folinic acid combined with a Phe-restricted diet (Table 3). Mildly affected individuals may not require any treatment [72]. 

### 4.4. PCD Deficiency

The deficiency of PCD (OMIM #264070) causes a mild form of HPA in newborns [73]. So far, 30 patients with PCD are listed in the BIODEF (biopku.org, accessed on 15 March 2023), and a total of 32 gene variants have been found to be responsible for PCD deficiency [74]. Neopterin values are increased in the disease, ranging from 4.07 to 22.48 mmol/mol creatinine in the urine and from 46 to 117 nmol/L in the CSF. The BH4 loading test shows a reduction in plasma Phe concentrations after the administration of 20 mg/kg of BH4 [28].

Usually, patients with PCD deficiency show no major alterations in Phe and neurotransmitter levels and no significant clinical abnormalities other than transient alterations in tone, having an excellent prognosis [75]. Indeed, it has been proposed that unspecific enzymes might compensate for the deficiency later in life [76]. When needed, treatment includes a Phe-restricted diet, oral sapropterin dihydrochloride, neurotransmitter precursors (L-dopa/decarboxylase inhibitor), 5-hydroxytryptophan, and folinic acid (Table 3). 

**Table 3:** Tetrahydrobiopterin (BH4) biosynthesis defects with hyperphenylalaninemia (HPA).

Pterin Metabolism Defects with HPA						
Disease Name	OMIM	Gene	Affected Enzyme	Symptoms	Diagnostic Based on Metabolites in CSF	First Line Treatment
Autosomal recessive GTP cyclohydrolase I deficiency	233910	*GCH1*	GTPCH I	Developmental delay, hypotonia, hypertonia, dystonia, hypersalivation	↓ HVA ↓ 5-HIAA ↓ /N 5-MTHF ↓ Biopterin ↓ Neopterin N Sepiapterin	Phe-reduced diet Sapropterin dihydrochloride L-Dopa/DC inhibitor 5-Hydroxytryptophan Folinic acid
6-pyruvoyl-tetrahydropterin synthase deficiency	261640	*PTS*	PTPS	Developmental delay, hypotonia, hypertonia, epilepsy, cognitive impairment, low birth weight	↓ HVA ↓ 5-HIAA ↓ /N 5-MTHF ↓ Biopterin ↑ Neopterin N Sepiapterin	Phe-reduced diet Sapropterin dihydrochloride L-Dopa/DC inhibitor 5-Hydroxytryptophan Folinic acid
Q-dihydropteridine reductase deficiency	261630	*QDPR*	DHPR	Developmental delay, hypotonia, hypertonia, epilepsy, microcephaly	↓ HVA ↓ 5-HIAA ↓ /N 5-MTHF ↑ Biopterin ↓/N Neopterin N Sepiapterin	Phe-reduced diet L-Dopa/DC inhibitor 5-Hydroxytryptophan Folinic acid
Pterin-4-alpha-carbinolamine dehydratase deficiency	264070	*PCBD1*	PCD	Developmental delay, hypotonia, hypomagnesemia, MODY3-like diabetes	NR HVA NR 5-HIAA NR 5-MTHF N Biopterin N Neopterin N Sepiapterin	Phe-reduced diet Sapropterin dihydrochloride L-Dopa/DC inhibitor 5-Hydroxytryptophan Folinic acid

BH4 deficiencies with HPA are described in the table. Main clinical and biochemical alterations, as well as the available palliative treatment are indicated for each disease. Abbreviations: Phe: phenylalanine, DC: decarboxylase; HVA: homovanillic acid; 5-HIAA: 5-Hydroxyindoleacetic acid; 5-MTHF: 5-Methyltetrahydrofolate; ↓: decrease; ↑: increase; N: normal; NR: not reported. Adapted from [77].

### 4.5. SPR Deficiency

The deficiency of SPR (OMIM #182125) was the last BH4 inherited metabolic disorder to be identified [78]. So far, 55 patients with SPR are listed in the BIODEF (biopku.org, accessed on 15 March 2023), 4 of them presenting the mild/peripheral subtype and 47 presenting as severe. Individuals with SPR deficiency do not have HPA; therefore, they are not detected by newborn screening, and the pterin levels in urine can be normal [78,79]. The absence of HPA is believed to occur due to the presence of alternative non-specific enzymes (such as CRs and/or ARs) that compensate for the deficient activity of SPR [28]. Normal to slightly increased levels of neopterin can be found in the CSF (Table 4) [28].

SPR deficiency is a genetic disease with autosomal recessive inheritance [78]. Patients with SPR deficiency can present in infancy only with severe developmental delay, which is followed by typical symptoms of a neurotransmitter defect: inconsolable crying, hypotonia, tremor, and ataxia, with diurnal variations. Psychiatric manifestations are common [80,81]. So far, 104 reported pathogenic variants distributed over the entire SPR gene have been reported [74]. Treatment includes the administration of oral sapropterin dihydrochloride combined with L-DOPA/decarboxylase inhibitor, and 5-hydroxytryptophan (Table 4). 

**Table 4:** Tetrahydrobiopterin (BH4) biosynthesis defects without hyperphenylalaninemia (HPA).

Pterin Metabolism Defects without HPA						
Disease Name	OMIM	Gene	Affected Enzyme	Symptoms	Diagnostic Based on Metabolites in CSF	First Line Treatment
Autosomal dominant GTP cyclohydrolase I deficiency	600225	*GCH1*	GTPCH I	Hypertonia, diurnal fluctuation of symptoms, dystonia, gait difficulties, hyperreflexia	↓ /N HVA ↓ /N 5-HIAA NR 5-MTHF ↓ /N Biopterin ↓ /N Neopterin N Sepiapterin	Sapropterin dihydrochloride L-Dopa/DC inhibitor
Sepiapterin reductase deficiency	182125	*SPR*	SPR	Developmental delay, hypotonia, hypertonia, cognitive impairment, impaired speech development, dysarthria, diurnal fluctuation of symptoms, dystonia, oculogyric crises, dyskinesia/other involuntary movements, hypokinesia, hypersalivation, psychiatric and sleep problems	↓ HVA ↓ 5-HIAA N 5-MTHF ↑ Biopterin N Neopterin ↑ Sepiapterin	Sapropterin dihydrochloride L-Dopa/DC inhibitor 5-Hydroxytryptophan

BH4 deficiencies without HPA are described in the table. Main clinical and biochemical alterations, as well as the available palliative treatment are indicated for each disease. Abbreviations: DC: decarboxylase; HVA: homovanillic acid; 5-HIAA: 5-Hydroxyindoleacetic acid; 5-MTHF: 5-Methyltetrahydrofolate; ↓: decrease; ↑: increase; N: normal; NR: not reported. Adapted from [77].

## 5. New Fundamental Roles of BH4 Metabolism Denoting Its Multifaceted Biological Functions 

New fundamental roles for BH4 metabolism have recently been uncovered. Our group has described the physiological properties for the BH4 pathway as having antioxidant and anti-inflammatory activities (inhibits the assembly and activation of the inflammasome) and being a mitochondrial activator, as well as a memory enhancer in the nervous system [7,17,18,82,83]. In addition, we have demonstrated that the genetic or pharmacological inactivation of *Gch1* impairs T cell proliferation and tissue infiltration, by a mechanism that involves iron homeostasis and mitochondrial activity in animal models of diseases with chronic inflammation, which was further confirmed in human cells [83].

Conversely, we have also demonstrated that exacerbated activation of the BH4 pathway is pathogenic, causing mitochondrial dysfunction, increasing the aggressiveness of the immune system, inducing pain hypersensitivity, and the progression of symptoms of chronic diseases, including chronic pain, asthma, multiple sclerosis, ulcerative colitis, rheumatoid arthritis, cognitive impairment, and others [83,84,85,86,87].

Our group has also shown that pain clinical scores can be attenuated or normalized if the physiological metabolic flux of the BH4 pathway is reestablished. This effect was observed in various animal models for chronic pain [84]. The discovery of this mechanism opened new and innovative therapeutic horizons to treat chronic inflammatory conditions safely and efficiently, based on the inhibition of SPR [83,84,85]. The use of specific inhibitors for BH4 synthesis called SPRi3 and QM385, reduced the BH4 levels in sensory neurons in active pain, eliciting analgesia [83,84,85]. Also, the identification of increased levels of sepiapterin in biological fluids as a marker of pharmacological BH4 metabolism engagement, offered for the first time, a quantitative and independent measure of analgesia [88]. 

## 6. Biological Effects of Neopterin

There is vast literature exploring the potential mechanistic toxic roles of neopterin, due to its association with acute and chronic inflammation (more than 3500 publications in the last five decades). In general, these studies have shown increased translocation of the nuclear factor kappa B (NF-kB), increased intracellular calcium, reactive oxygen species (ROS) production, oxidative stress, mitochondrial dysfunction, increased proto-oncogene expression, apoptosis, and reduced cell viability in a variety of human and rodent cells exposed to neopterin [89,90,91,92,93,94]. All these mechanisms have already been implicated in the physiopathology of multiple inflammatory states, including obesity and obesity-related comorbidities, such as cardiovascular diseases, type 2 diabetes mellitus, atherosclerosis, hypertension, and musculoskeletal and neurodegenerative diseases. In fact, our group has also demonstrated increased neopterin in individuals affected by diabetic neuropathic pain [86] and in acute-on-chronic liver failure patients [95]. However, information regarding the functional physiological activities of neopterin is scarce. 

Neopterin has been reported to be a silent and inert by-product of BH4 metabolism, acting as a marker for activation of the innate immune response. However, our group has described new activities for neopterin under non-inflamed physiological conditions. For example, we demonstrated increased resistance to induced oxidative stress in the mouse brain after a single intracerebroventricular (i.c.v.) administration of neopterin (4 pmol), which generated slightly increased neopterin levels in the CSF [17,82]. The experimental treatment increased glutathione, glutathione-metabolizing enzyme activity, respiratory chain complex I/IV activity, and basal oxygen consumption, but reduced glycolysis, through activation of the nuclear factor-erythroid factor 2-related factor 2 (Nrf2)/antioxidant response element (ARE) pathway [17,82]. All these positive effects of neopterin might have contributed to the mnemonic effect previously demonstrated [96]. Neopterin administration (i.c.v.) enhanced learning and memory by facilitating long-term potentiation (LTP) [96], which represents the acquisition and maintenance of memories at the synaptic level [97].

**Figure 3:**
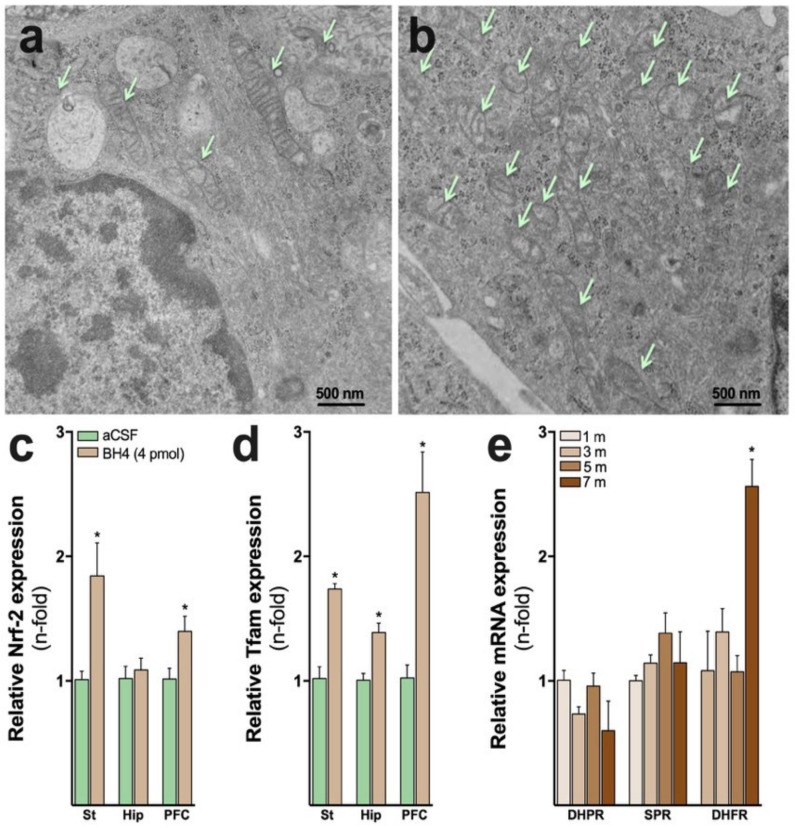
Tetrahydrobiopterin (BH4) pathway in the brain of naïve mice and the effect of its metabolites on mitochondrial parameters in sensory neurons. (**a**,**b**) Increased mitochondrial number in dorsal root ganglia neurons (sensory neurons) exposed to 50 nM neopterin for 24 h. (**b**) Arrows denote increased organelle number. Bars indicate 500 nm. Electron microscopy micrographs: 10,000× magnification. (**c**,**d**) Increased Nrf-2 and Tfam expression in different brain regions after 24 h of a single intracerebroventricular injection of 4 pmol BH4 (1 μL) in C57Bl6 mice. St: striatum; Hip: hippocampus; PFC: prefrontal cortex; aCSF: artificial cerebrospinal fluid. (**e**) Expression of the genes coding dihydropteridine reductase (DHPR; BH4 recycling pathway), dihydrofolate reductase (DHFR; BH4 salvage pathway), and sepiapterin reductase (SPR; BH4 de novo and salvage pathways) in the hippocampus of naïve C57Bl6 mice. 1, 3, 5, and 7 m: 1, 3, 5 or 7-month-old mice. * *p* < 0.05.

Similar antioxidant functions were induced in cell-based systems, including primary rat astrocytes, C6 astroglial cells, and human nerve cells, when exposed to very low concentrations of neopterin [17,82]. In addition, an increased mitochondrial number was observed in neopterin-treated primary dorsal root ganglion (DRG) neurons (sensory neurons), indicating that mitochondrial fusion–fission processes or biogenesis are induced or maintained by physiological levels of neopterin. Figure 3a–d shows the positive effects of boosting neopterin levels on mitochondrial content and the expression of Nrf-2/ARE-related genes in the DRG neurons and mouse brain. Figure 3 shows that the single exposure (24 h) of sensory neurons to neopterin increased the number of mitochondria (Figure 3b) when compared to the controls (Figure 3a), suggesting a boost in energy metabolism. In agreement, the i.c.v. administration of neopterin (4 pmol) increased the expression of Nrf-2 (Figure 3c) and Tfam (Figure 3d), which suggest increased antioxidant defenses and mitochondrial health. In this scenario, Nrf-2 is a master transcription factor that finely regulates the cellular antioxidant response [98,99]. The Nrf2/ARE system regulates the transcription of approximately 250 genes, including antioxidants crucial for cellular redox control in the brain and skeletal muscle, tissues highly dependent on mitochondrial activity for energy synthesis. Some of the modulated genes encode for the antioxidant enzymes superoxide dismutase, glutathione peroxidase, glutathione reductase, hemeoxygenase type 1 (HO-1), peroxiredoxin, thioredoxin reductase, thioredoxin, and metallothionein. The increase in the protein content of these enzymes has been correlated with several beneficial effects in tissues with high mitochondrial content (for a review see [100]). Furthermore, Tfam is a protein encoded by TFAM that performs multiple functions of transcriptional activation and organization of mitochondrial DNA (mtDNA) [101,102]. Tfam is also necessary for the regulation of enzymes involved in oxidative phosphorylation, as well as for the maintenance of mtDNA, thus playing a role in the organization of the mitochondrial genome [102]. These novel data suggest that neopterin at physiological concentrations might serve as a key metabolite-activating antioxidant and energy pathways that can promote cell survival. Crosstalk among metabolic routes to promote cellular homeostasis has already been demonstrated for other metabolic intermediates, e.g., NAD+ [103]. It has been extensively demonstrated that the NAD+ pathway functions as a critical regulator to maintain physiologic processes, enabling cells to adapt to environmental changes, including nutrient perturbation, genotoxic factors, circadian disorder, infection, inflammation, and xenobiotics [103].

## 7. Biological Effects of BH4

Considering that neopterin is a byproduct of the de novo BH4 pathway and that its levels reflect the magnitude of GTPCH activity, similar intracellular effects should be expected for BH4 under non-inflammatory conditions. Indeed, positive effects on the antioxidant system, mitochondrial physiology, and dynamics and cognition were observed in the brain and the immune system of animals with enhanced BH4 metabolism [18,83]. 

As previously shown for neopterin, a single i.c.v. administration of BH4 enhanced the hippocampal cognition via a mechanism linked to the activation of glutamatergic neurotransmission and cell threshold reduction, which facilitated LTP triggering in various rodent strains [18]. In agreement, it has been reported that BH4 administration in PKU-affected patients (a hereditary inability to transform Phe into Tyr) improved working memory and brain activation [104].

The antioxidant activities of BH4 might have also contributed to the observed mnemonic effect. In fact, it has been demonstrated that BH4 is a more reactive scavenger of superoxide, hydroxyl and thiyl radicals, and peroxynitrite than ascorbate at the physiological blood pH [105,106]. It has also been proposed that BH4 would enhance mitochondrial activity when thiol compounds, such as glutathione are reduced [107], suggesting the existence of a mitochondrial BH4 pool. Moreover, it was shown experimentally that *Gch1* (gene encoding the rate-limiting enzyme of BH4 biosynthesis) is a direct target of Nrf-2 in skin cells submitted to radiation (Nrf-2 has a binding site in the proximal promoter of the *Gch1* gene), and the resultant increased intracellular BH4 levels promoted cytoprotection by neutralizing ROS [108]. This is in agreement with previous results showing neopterin-induced Nrf-2 nuclear translocation with increased content of key cytoprotective downstream proteins, including the antioxidant HO-I (it metabolizes heme into biliverdin, a potent antioxidant) in rodent and human cells [17]. It has also been shown that immune cells lacking BH4 production showed increased content of iron-related proteins, including mitoferrin, ferritin, and frataxin, all mitochondrial proteins [83], as a potential compensatory effect.

It is also known that BH4 autooxidation can be induced by a variety of biochemical reactions, including the interaction with ferricytochrome *c* with the production of BH2 via the formation of an intermediate radical [109,110]. In this scenario, it was shown that BH4 efficiently reduces ferricytochrome *c* to ferrocytochrome *c* (transition between complexes III and IV of the respiratory chain) at physiological BH4 levels in activated T cells. Thus, ablated mouse *Gch1* T cells impaired mitochondrial respiration, which was improved by the replenishment of BH4 with sepiapterin, or by delivering ferrocytochrome *c* directly into the mitochondria [83]. The data presented in Figure 3d also shows that BH4 enhanced the expression of Tfam (a mitochondrial transcription factor involved in the genesis of new mitochondria) in the mouse brain. Increased Tfam was also demonstrated to be induced by BH4 in the heart, in addition to peroxisome proliferator-activated receptor gamma coactivator-1 alpha (PGC-1α) and estrogen-related receptor alpha, which are nuclear transcription factors that cooperate with Tfam for mitochondrial biogenesis [111].

The oxidation of BH4 will compromise the BH4/BH2 ratio, which is determinant for proper NOS activity and, therefore, the production of NO. BH2 serves no cofactor actions and binds to NOS with approximately the same affinity as its reduced form. Under these conditions, NOS activity becomes uncoupled resulting in the production of the superoxide radical and ONOO^−^ [48], toxic reactive compounds that rapidly oxidize BH4 favoring the establishment of oxidative stress [105,106] (Figure 2b). Uncoupled NOS has already been described in a variety of experimental and clinical chronic conditions; namely type 2 diabetes mellitus, hypertension, cigarette smoke-induced lung dysfunction, heart failure, atherogenesis, chronic pain, and neurodegenerative diseases, which are all comorbidities of chronic diseases. Given the intrinsic BH4 deficiency/NOS uncoupling relationship, many clinical studies have focused on pharmacological interventions to enhance BH4 bioavailability to attenuate vascular dysfunction in chronic conditions [112,113,114,115].

Due to the positive intracellular effects of BH4, its deficiency may be considered a risk factor for the development of chronic diseases, including obesity, aging, vascular disorders, neurodegenerative diseases, and others, characterized by inflammation, oxidative stress, and mitochondrial dysfunction. In agreement, Figure 3e depicts increased DHFR expression in the hippocampi of naïve 7-month-old mice, phenomenon that might represent a compensatory mechanism to maintain BH4 at physiological levels during natural aging. In agreement, reduced BH4 levels have been reported in the CSF, blood and postmortem brain of individuals affected by common neurodegenerative diseases [116,117]. 

BH4 levels have also been proposed as being required for normal brain maturation. For example, data generated from experimental studies showed the association of reduced BH4 levels and brain maturation delay in a mouse model for prenatal hypoxia in congenital heart disease [118]. In agreement with various other studies, exogenous BH4 administration resulted in enhanced sensorimotor coordination, normalizing the delay in myelination due to hypoxia, and decreasing white matter apoptosis during brain development. In addition, rabbits exposed to hypoxia-ischemia showed reduced neuronal death in the cortex, basal ganglia, and thalamus, suggesting that BH4 deprivation in premature brains may be a risk factor for survival [119].

## 8. GTPCH and SPR Deficiencies Affect Energy Metabolism

Emerging evidence obtained from multiple animal models depicting energy depletion in tissues with high mitochondria content has shown that BH4 deficiency compromises mtDNA transcription, mitochondrial biogenesis, and respiration. In agreement, it was shown that T cell physiology is also dependent on BH4 appropriate concentrations [83]; thus, it seems feasible to propose that the dependency of mitochondrial health on BH4 synthesis is a physiological path of tissues with high energy demands. For example, low levels of BH4 have been associated with a broad range of cardiovascular and metabolic diseases, including hypertension, hypertrophy, ischemic heart disease, and diabetes mellitus, in animal models and human patients [120,121,122]. Indeed, it has been shown that several metabolic pathways and proteins are modulated in a mouse heart when the synthesis of BH4 is compromised. A systems-based integrative data analysis to investigate systematic changes in the cardiac mitochondrial proteome of SPR-null mice demonstrated specific nodes in a pathway–pathway network that involved compromised energy production, and lipid, carbohydrate, and amino acid metabolisms. This analysis also showed a specific protein–protein network with compromised content of oxidative phosphorylation proteins. In agreement, reduced oxygen consumption (a measure of mitochondrial physiology), impaired ATP synthesis with loss of mitochondrial membrane potential, and increased ROS formation were confirmed in the hearts of SPR-null mice [111]. As expected, the remodeled bioenergetics and the oxidant environment reduced cardiac function and decreased life expectancy. However, the restitution of BH4 to normal levels (20 mg/kg/day, i.p.; 2 weeks) rescued the mitochondrial activity, attenuated ROS production, and normalized cardiac systolic function, along with body size and weight [111]. These effects were mostly linked to the positive regulation of PGC-1α [111], a coactivator of transcription needed for mitochondrial biogenesis [123]. In fact, BH4 deficiency impaired the expression of the PGC-1α-dependent genes that regulate mitochondrial biogenesis, mtDNA transcription, and mRNA translation [111], suggesting a prominent role of BH4 metabolism in mitochondrial biogenesis.

It has also been shown that BH4 deficiency, due to the lack of SPR activity, resulted in impaired mitophagy, a process required to maintain the quality and quantity of healthy mitochondria [124]. Dysregulation of mitochondrial quality control was evident in the brain, liver, muscle, and lung of SPR-null mice, and it was counterbalanced when BH4 intracellular concentrations were normalized [125]. The underlying molecular mechanisms compromising mitophagy were proposed as being linked to limited availability of Tyr, a semi-essential amino acid, whose synthesis is dependent on BH4 production. In agreement, human PKU-derived lymphocytes showed a high Phe/Tyr ratio with insufficient mitophagy [125], revealing an intimate relationship between BH4 deficiency and impaired autophagy.

The effects induced by the deficiency of BH4 have also been characterized in cells that are fully dependent on mitochondrial activity to generate energy. The pharmacological inhibition of GTPCH by using the selective and direct-acting inhibitor 2,4-diamino-6-hydroxypyrimidine resulted in oxidative stress, mitochondrial depolarization, ATP depletion, inhibition of complex IV, and necrosis in cortical neurons submitted to hypoxia [126]. All these effects were counteracted when neuronal BH4 levels were restored, by incubating cells with exogenous BH4 during the hypoxic period. Similarly, hypoxia-induced damage in BH4-deficient neurons was prevented when a NOS inhibitor, hemoglobin, or superoxide dismutase plus catalase were present during the hypoxic period [126], suggesting that NOS uncoupling might be involved in deficient energy metabolism. From the data presented, it can be concluded that energy metabolism requires appropriate intracellular levels of BH4 to support bioenergetic processes that are independent of those catalyzed by BH4 as an enzyme cofactor.

## 9. Non-BH4-Linked Genetic Deficiencies of BH4 Metabolism

### 9.1. ASD

ASD is a disorder that describes individuals who have persistent deficits in social communication and social interaction with restricted, repetitive patterns of behavior, interest, or activities. ASD represents a large spectrum of classifications and presentations, from mild to severe impairment. ASD’s physiopathology is not very well understood; however, evidence from the literature suggests a specific modulation of the BH4 pathway affecting the activity of the monoaminergic neurons that might be downregulated in the disease. Indeed, reduced levels of BH4 have been demonstrated in the blood, urine, and CSF of ASD-affected children [59,127]. Additionally, several reports have demonstrated a therapeutic effect of BH4 supplementation in children with ASD. BH4 daily doses of 1 to 3 mg/kg for 4 to up to 105 weeks elicited marked improvements in social responsiveness, communication, and cognitive abilities in over 300 mildly to severely affected ASD children [128,129,130,131,132,133,134]. These improvements were marked in children with higher intelligence and younger than 5 years old [128,132]. Interestingly, BH4 daily supplementation with 20 mg/kg induced similar effects than lower doses [60,114,128].

The low levels of BH4 in young children with ASD has been proposed to be related to high NO production in response to excessive inflammation and overactivation of the immune system [135]; folate deficiency, which will impact negatively on the BH4 salvage pathway activity through the participation of DHFR [136,137]; and excessive oxidative stress that will consume the antioxidant BH4 [18,138].

The involvement of the BH4 pathway was also suggested in a study that investigated 247 patients presenting autism, who were referred from pediatric-psychiatric to pediatric- metabolic outpatient clinics. Six patients from this group were affected by different metabolic disorders, and one of them by PKU [139]. In addition, genome-wide studies showed a significant nominal association in the *PTPS* gene with ASD [140]. Furthermore, a spontaneous copy number variation in the *AGMO* gene, leading to a deletion within exons 2–8, was identified in a patient with ASD [56]. Another study demonstrated that de novo mutations within the *AGMO* gene were involved in ASD [57].

### 9.2. Human Rabies

Reduced levels of BH4 and BH4-dependent metabolites have been demonstrated in human rabies [141]. In agreement, oral BH4 supplementation has been shown to be responsible for a rapid increase in the CSF concentrations of BH4 and the neurotransmitters dopamine and serotonin, resulting in increased survival chances for patients affected by rabies [142]. These findings strongly suggest that the neurochemical dysfunction observed in patients infected with rabies virus is, at least in part, due to impaired BH4 intracellular concentrations.

### 9.3. Cerebral Malaria

Impaired BH4 metabolism has also been identified in cerebral malaria, having the lowest CSF levels of BH4 when the affected individuals were in a deep coma. In addition, elevated CSF neopterin, its levels were positively associated with the duration of fever before coma, indicating that inflammation and the consequent induction of oxidative stress may be responsible for reduced BH4 levels [143]. In turn, low BH4 concentrations compromise effector immune function, contributing to coma [144]. Again, this agrees with previous studies showing that appropriate BH4 levels are needed for immune cell physiology [83]. 

### 9.4. PD

It has been extensively proposed that low levels of BH4 might be linked to the impaired dopaminergic neurotransmission in this neurodegenerative disease. High gene expression of *SPR* has been identified in the postmortem brains of patients affected by PD [116], pointing to a compensatory and protective mechanism due to the reduction in BH4 levels. In agreement, SPR has been proposed as a protective factor in neurons against methyl-4-phenylpyridinium-induced toxicity [145]. On the other hand, the expression of AR and PTS genes and CSF BH4 concentrations were decreased in brains affected by PD, indicating compromised BH4 metabolism and, consequently, impaired dopaminergic metabolism and neurotransmission [116,146,147]. In agreement, we have shown that the administration of levodopa to rodents increases the availability of BH4 in the striatum [148]. Furthermore, when levodopa was supplemented in mice submitted to an experimental model of PD, the levels of BH4 were rescued, suggesting that part of the effects mediated by this pharmacological treatment involves the restauration of the levels of BH4 metabolism. Indeed, this approach stimulated the BH4 salvage pathway by upregulating DHFR expression [148].

### 9.5. Alzheimer’s Disease (AD)

AD is the most common form of neurodegenerative disease, estimated to contribute 60–70% of all the cases of dementia worldwide [149]. According to the prevailing amyloid cascade hypothesis, amyloid-β deposition in the brain is the initiating event in AD, although evidence is accumulating that this hypothesis is insufficient to explain many aspects of AD pathogenesis. The interplay among mitochondrial dysfunction, inflammation, and oxidative stress has extensively been proposed as key factors of its physiopathology. Therefore, it could be expected that the levels of BH4 under this scenario are compromised. In fact, BH4 metabolism (enzymes and metabolites) was demonstrated to be reduced in several brain regions of postmortem histologically confirmed AD cases [117,150]. Additionally, the administration of BH4 rescued memory impairment in 13-month-old 3×Tg-AD mice, without having any impact on the neuropathology [151], suggesting that the memory deficit may be linked to BH4 metabolism. The evidence demonstrating reduced BH4 availability in AD is the basis for the ongoing clinical trial based on the off-label (drug repurposing) use of BH4 (ClinicalTrials.gov, accessed on 15 March 2023).

### 9.6. Fabry Disease

The inborn error of metabolism known as Fabry disease is caused by the deficient activity of α-galactosidase-A and the, subsequent, accumulation of glycosphingolipids (mainly globotriaosylceramide, Gb3) [152]. The disease is characterized by a variety of clinical manifestations, including acroparesthesias, angiokeratomas, stroke, hypertrophic cardiomyopathy, and progressive renal impairment [153]. Enzyme replacement therapy is currently the standard of care for symptomatic Fabry patients, but its physiopathology is not well understood. It was recently demonstrated that BH4 is decreased in the plasma of female Fabry patients, which was not corrected by enzyme replacement therapy. When the metabolism was investigated in the animal model of the disease, the Fabry mouse, BH4 was confirmed to be decreased in the heart and kidney, but not in the liver and aorta. Moreover, Gb3 levels were inversely correlated with BH4 levels in animal tissues and cultured patient cells, pointing out that the accumulation of the metabolite is favored by the oxidation of BH4. In agreement, the administration substrate reduction therapy restored the levels of BH4 and the clinical phenotype. Additionally, the intervention rescued markers of oxidative stress present in the experimental model, e.g., the levels of glutathione were recovered, increasing the antioxidant defenses of the cell.

## 10. BH4 Administration as a New Therapeutic Horizon for Mitochondrial Diseases

Mitochondriopathies are disorders characterized by defects in the mitochondrial structure and function due to mutations, depletion, or deletions of nuclear DNA and/or mtDNA (mtDNA) [154]. Changes in the mitochondrial structure and function can compromise the functioning of the mitochondrial respiratory chain, reduce energy production, alter the cellular redox state, increase the production of ROS, deregulate calcium homeostasis, and induce apoptosis, eliciting mtDNA destabilization (for a review see [155]). At present, there is no cure and only supportive and symptomatic therapies are available. Obstacles to finding effective treatments include the rarity of the disease, clinical diversity, genetic heterogeneity, difficult clinical trials, and poorly understood pathophysiology.

Based on our knowledge about the non-canonical biological roles of BH4 metabolism and preclinical studies from the last decade, we hypothesize that BH4 supplementation might be an innovative and safe way to treat disorders whose physiopathology involves the interplay of mitochondrial dysfunction, oxidative stress, and inflammation. Given that BH4 seems to be essential for proper mitochondrial activity and antioxidant activation in tissues with high energy demands, it is plausible that BH4 supplementation might represent an effective strategy to increase residual mitochondrial function in genetic mitochondrial diseases. 

There is evidence in the literature, generated from clinical and basic studies, demonstrating that impaired mitochondrial function, and increased oxidation and inflammation are also common pathological mechanisms in hereditary metabolic disorders [156,157,158,159,160,161,162]. Thus, by increasing BH4 intracellular levels, it could be expected that key antioxidant, anti-inflammatory, and mitochondrial-related functions would be enhanced, for example, in the brain [7,17,18,96,163]. Although this approach might not represent the cure for primary mitochondrial disorders, it has the potential to alleviate disease burden and improve the quality of life for affected individuals and their families.

BH4 is sold commercially, as sapropterin dihydrochloride, in tablets or powder for oral solution formulations, and it is available in 63 countries around the world. Sapropterin dihydrochloride was approved on 13 December 2007 by the U.S. Food and Drug Administration (FDA) in 2007 to be used in conjunction with a Phe-restrictive diet to reduce blood Phe levels in patients with HPA due to BH4-responsive PKU (www.accessdata.fda.gov, accessed on 15 March 2023; application number ND 022181). Since then, more than 7800 patients (including 1560 children under 4 years of age) have been treated with sapropterin dihydrochloride in the United States. Several clinical trials have shown the compound is safe and well tolerated [164]. The recommended doses to reduce HPA or to increased Phe tolerance have ranged between 1 to 20 mg/kg [67].

**Figure 4:**
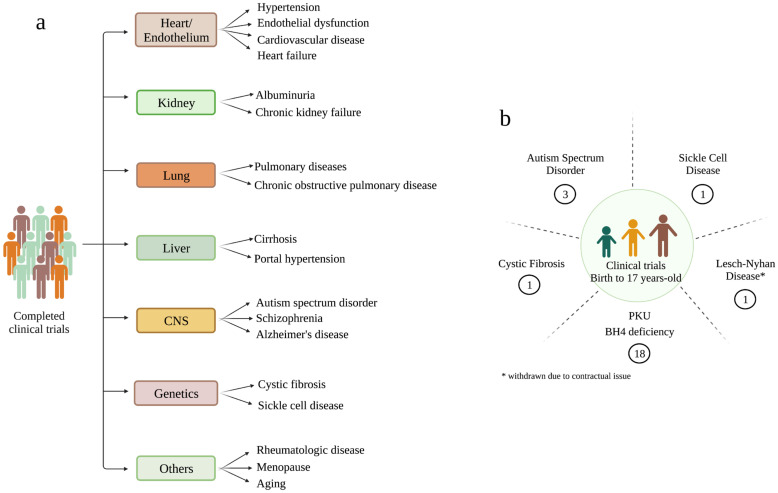
Food and Drug Administration (FDA)-approved clinical trials for the off-label use of tetrahydrobiopterin (BH4) for a variety of disorders not primarily affecting phenylalanine (Phe) metabolism. (**a**) Completed clinical trials where BH4 was administered to individuals affected by heart and vascular diseases, kidney, lung, liver, central nervous system (CNS), and genetic disorders. Other miscellaneous conditions were also included in the clinical trials, such as rheumatologic diseases, menopause, and aging. (**b**) Clinical trials involving the administration of BH4 in children affected by genetic disorders. Circled numbers correspond to the number of clinical trials based on BH4 administration. * The study was withdrawn before enrolling its first participant, due to contractual issues.

According to the FDA, the use of an approved drug for an unapproved use, often called “off-label” use, is justified if it is judged to be medically appropriate to treat a particular condition. In this scenario, the off-label use of sapropterin dihydrochloride has grown during the last two decades, supporting clinical trials for cardiac, pulmonary, rheumatologic, dermic, and psychiatric diseases, dementia, menopause, aging, and inherited disorders (Figure 4a). 

All these clinical trials are published on ClinicalTrials.gov and were based on the safety of sapropterin dihydrochloride administration [164], and the large body of information generated from preclinical studies supporting the potential benefits induced by BH4 supplementation. Detailed information about the clinical trials aimed at identifying the beneficial effects of BH4 administration on a variety of diseases can be found at www.ClinicalTrials.gov.

The physiopathology of the abovementioned disorders is still not fully defined; however, there is a clear consensus that mitochondrial dysfunction, sustained inflammatory responses, and increased oxidative stress, play a role in their onset and development. Additionally, it has been proven that the supplementation with antioxidants (e.g., ascorbic acid, vitamin E), mitochondrial activators or energy substrates (e.g., creatine, lipoic acid, coenzyme Q), and anti-inflammatory drugs (e.g., sulfasalazine, biologicals) may decrease the associated mortality and morbidity. Therefore, it seems plausible to propose the off-label use of sapropterin dihydrochloride supplementation for hereditary metabolic disorders linked to energy production. 

## 11. Conclusions

BH4 metabolism has emerged in the last decade as a promising metabolic target to modulate toxic pathways that may accelerate cell death. Strong evidence generated by our group and others has shown that BH4 metabolism has multiple biological roles, supporting essential pathways that generate energy, enhance antioxidant resistance, and protect against sustained inflammation. Therefore, BH4 should not be understood solely as an enzyme cofactor, and neopterin should not be considered only as an inert byproduct of BH4 metabolism. 

**Figure 5:**
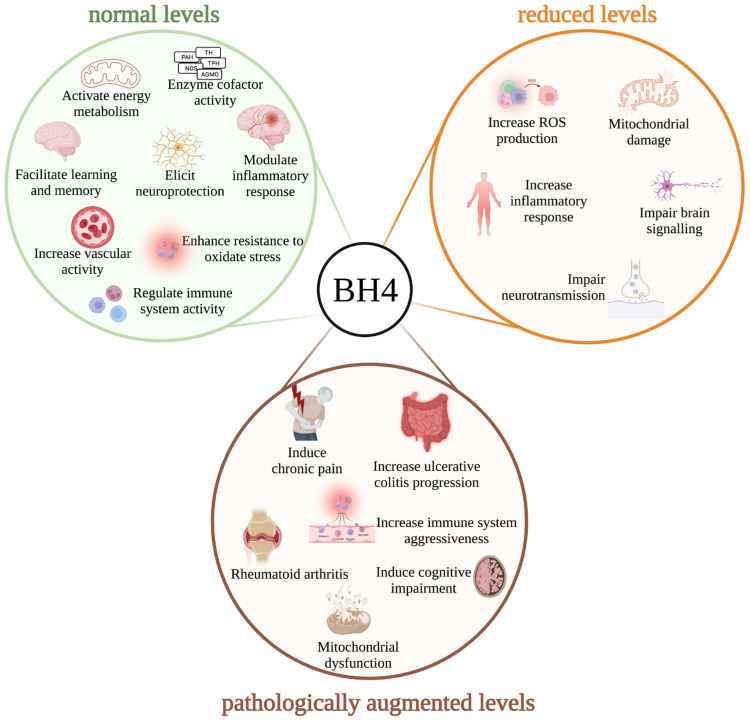
Tetrahydrobiopterin (BH4) metabolism as a central hub regulating physiological and toxic pathways. **Normal BH4 levels** (green circle): Physiological levels of BH4 sustain the traditional coenzyme activity of the pathway, favoring the correct metabolism of aromatic amino acids and ether lipids, and the biosynthesis of nitric oxide. Under these conditions, appropriate BH4 levels activate energy metabolism, enhance cellular resistance to oxidative stress, modulate the inflammatory response, facilitate learning and memory, regulate immune system activity, increase vascular activity, and exert neuroprotective effects. **Reduced BH4 levels** (orange circle): When BH4 levels are perturbed, ATP synthesis and brain lipid signaling are impaired, an oxidant status is induced, neurotransmission is compromised, and inflammation is favored. **Pathologically augmented BH4 levels** (brown circle): Excessive intracellular BH4 levels induce mitochondrial dysfunction, compromise memory and learning, increase the aggressivity of the immune system, promote the progression of inflammatory and autoimmune diseases, and elicit chronic pain. Thus, BH4 metabolism can be considered a double-edged sword: too little or too much results in cytotoxicity.

BH4 metabolism can instead be depicted as a cytoprotective pathway that is finely regulated by the concerted action of de novo, salvage, and recycling pathways to regulate intracellular concentrations of BH4 and BH4-related metabolites. If this finely tuned balance in BH4 concentration is perturbed, several biological systems are compromised, resulting in impaired neurotransmission, immune responses, metabolism, and vascular activity (Figure 5). Correction of the imbalance resulted in improved cell homeostasis and survival. All these imbalances can potentially be attenuated by BH4 supplementation and could be beneficial in n mitochondrial disorders. However, it should be stressed that abnormally high intracellular levels of BH4 do not promote cytoprotection. On the contrary, we have shown, in human and experimental chronic diseases, that clinical presentation is worsened when levels of BH4 surpass therapeutic or physiological levels (Figure 5). Indeed, when an inhibitor of SPR was used to normalize BH4 levels, symptoms were attenuated. Thus, BH4 metabolism can be considered a double-edge sword, with too little or too much resulting in toxicity.

## Figures and Tables

**Table 1:** Reference values for tetrahydrobiopterin (BH4) and related metabolites.

	CSF			Urine	
Age	BH4 (nmol/L)	Biopterin (nmol/L)	Neopterin (nmol/L)	Biopterin (mmol/mol creatinine)	Neopterin (mmol/mol creatinine)
Newborns	25–121	20–70	15–35	0.5–3.0	1.1–4.0
0–1 year	24–59	15–40	12–30	0.5–3.0	1.1–4.0
2–4 year	20–61	10–30	9–20	0.5–3.0	1.1–4.0
5–10 years	20–49	10–30	9–20	0.5–3.0	1.1–4.0
11–16 years	20–49	10–30	9–20	0.5–2.7	0.2–1.7
		**Serum**		**Dried blood spot**	
		**Biopterin** (nmol/L)	**Neopterin** (nmol/L)	**Biopterin** (nmol/L Hb)	**Neopterin** (nmol/g Hb)
All ages		4–18	3–11	0.08–1.20	0.19–2.93

Cerebrospinal fluid (CSF), serum, urine, and dried blood spot BH4 levels, by age group. Adapted from [12].

**Table 2:** Neopterin levels in biological fluid from adults.

Neopterin Levels	
Age	CSF (nmol/L)
19–75	4.2 ± 1.0
Age	**Urine** **(μmol/mol creatinine)**
19–25	125 ± 32
26–35	112 ± 33
36–45	124 ± 33
46–55	126 ± 34
56–65	137 ± 37
>65	142 ± 39
Age	**Serum** **(nmol/L)**
19–75	5.3 ± 2.7
>75	9.7 ± 5.0

Adapted from [13].

## Data Availability

The data presented in this study are available in the article.
